# Rapid Diffuse Leptomeningeal Dissemination of an Anaplastic Pleomorphic Xanthoastrocytoma in an Adult Patient

**DOI:** 10.7759/cureus.19186

**Published:** 2021-11-01

**Authors:** Michael Bounajem, Kyril L Cole, Sarah T Menacho

**Affiliations:** 1 Neurosurgery, University of Utah, Salt Lake City, USA

**Keywords:** neurosurgery, craniospinal dissemination, anaplastic, brain tumor, leptomeningeal dissemination, anaplastic pleomorphic xanthoastrocytoma

## Abstract

Although pleomorphic xanthoastrocytomas generally carry a fair prognosis, anaplastic transformation has been identified in a subset of cases.We present the case of a patient with primary anaplastic pleomorphic xanthoastrocytoma (APXA) that demonstrated rapid recurrence and diffuse leptomeningeal spread of disease three months postoperatively, causing severe visual impairment and acute ischemic strokes leading to death.We believe this is the fastest reported time to leptomeningeal dissemination and death from the initial diagnosis. Through this case, we show how anaplastic features can have a highly variable biological effect on disease progression. We believe earlier craniospinal imaging at the time of APXA diagnosis should be pursued to manage disease progression more aggressively with currently recommended adjuvant therapies.

## Introduction

Pleomorphic xanthoastrocytomas (PXAs) are an uncommon primary central nervous system neoplasm, comprising >1% of astrocytomas [1]. They most commonly present as supratentorial nodulocystic tumors and disproportionately affect children and young adults. Although they generally carry a fair prognosis, anaplastic transformation has been identified in a subset of cases, though only rarely on initial presentation. Here we describe the rapid progression of leptomeningeal dissemination (LMD) of an anaplastic pleomorphic xanthoastrocytoma (APXA) in the right temporal lobe and discuss the prevalence, prognosis, and treatment options for this rare aggressive entity.

## Case presentation

**Figure 1:**
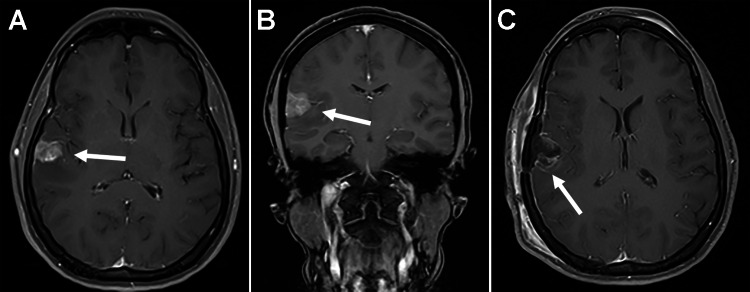
Magnetic resonance imaging (MRI) of the brain. (A) Axial and (B) coronal T1-weighted postcontrast MRI scans demonstrating a 1.3×1.8×1.7 cm right temporal mass. (C) Postoperative axial postcontrast MRI demonstrating gross total resection with no acute postsurgical complications and scant blood products in the resection cavity.

A 36-year-old woman with a past medical history of attention deficit hyperactivity disorder, asthma, and major depressive disorder presented with a 1.5-month history of unprovoked holocranial headaches as well as nausea. Magnetic resonance imaging (MRI) with and without contrast demonstrated a 1.3×1.8×1.7 cm enhancing right temporal mass with surrounding vasogenic edema (Figure 1 A and B). Differential diagnoses included PXA, ganglioglioma, and other glial neoplasms. Computed tomography (CT) scans of the patient’s chest, abdomen, and pelvis with contrast demonstrated no other source of malignancy. The patient underwent right temporal craniotomy for resection of the mass, and postoperative imaging demonstrated a gross-total resection (GTR) without evidence of acute postsurgical complication (Figure 1 C). Her postoperative course was uneventful, and she was discharged home on postoperative day two.

The initial pathology report identified a low-grade glioma with a BRAF V600E mutation. Further sequencing determined that the neoplasm was endothelial growth factor receptor nonamplified and O6-methylguanine DNA methyltransferase unmethylated. Subsequent methylation profiling demonstrated findings consistent with APXA, a World Health Organization (WHO) Grade III neoplasm. The patient was scheduled to proceed with adjuvant radiation therapy, but the treatment was delayed because she was admitted to a psychiatric hospital for worsening depression and anxiety.

**Figure 2:**
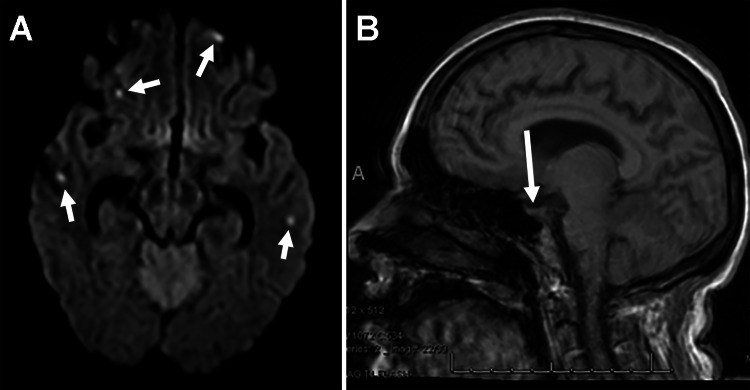
MRI obtained three months after initial diagnosis. (A) Axial diffusion-weighted imaging demonstrating diffuse punctate infarcts from leptomeningeal dissemination around numerous intracranial vessels. (B) Sagittal T1-weighted MRI scan demonstrating a flattened pituitary gland indicative of increased intracranial pressure.

Three months after the initial diagnosis, the patient re-presented to the emergency department with two weeks of progressive confusion, generalized weakness, and changes in her vision. She endorsed an inability to read or see color. The MRI with contrast demonstrated multiple punctate infarcts throughout the cerebral hemispheres in different vascular territories and a flattened pituitary, which was concerning for increased intracranial pressure (Figure 2). A lumbar puncture was performed with an opening pressure of 52 cm H20. Ophthalmology evaluation demonstrated bilateral Grade 4 optic disc edema with numerous retinal hemorrhages.

**Figure 3:**
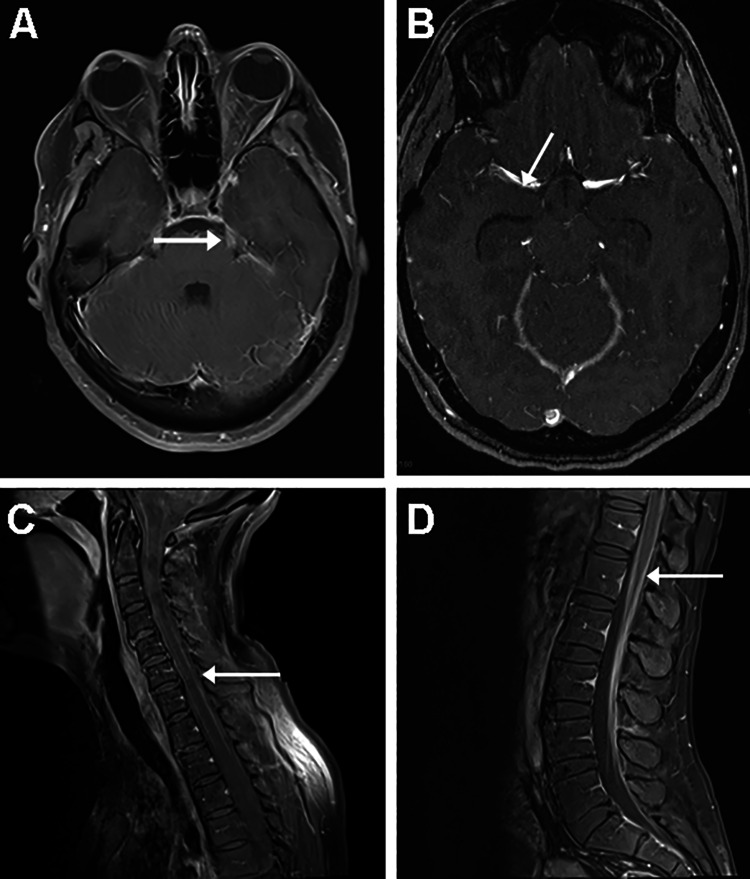
Repeat MRI of the brain Axial T1-weighted postcontrast MRI scans demonstrating (A) leftward midbrain/pontine enhancement and (B) vascular leptomeningeal enhancement around the right middle cerebral artery consistent with LMD. Sagittal postcontrast MRI of the (C) cervical and (D) lumbar spine demonstrating diffuse LMD of the tumor. LMD: Leptomeningeal dissemination

The patient underwent emergent placement of a right frontal external ventricular drain with an elevated opening pressure of 30 cm H20. Repeat MRI of the brain with and without contrast as well as MRI of the cervical, thoracic, and lumbar spine demonstrated diffuse leptomeningeal enhancement, including in the interpeduncular cistern, internal auditory canals, suprasellar cistern, bilateral oculomotor nerves, and bilateral trigeminal nerves and pial enhancement around the basilar artery and ophthalmic segments of the internal carotid arteries (Figure 3). In retrospect, the multiple strokes seen on the brain MRI, done when the patient returned to the hospital with LMD, were thus thought to be secondary to the vascular invasion of tumor-causing narrowing and ischemia. Additionally, cerebrospinal fluid analysis was negative for infection with cytology positive for malignant cells, confirming LMD of the tumor.

**Figure 4:**
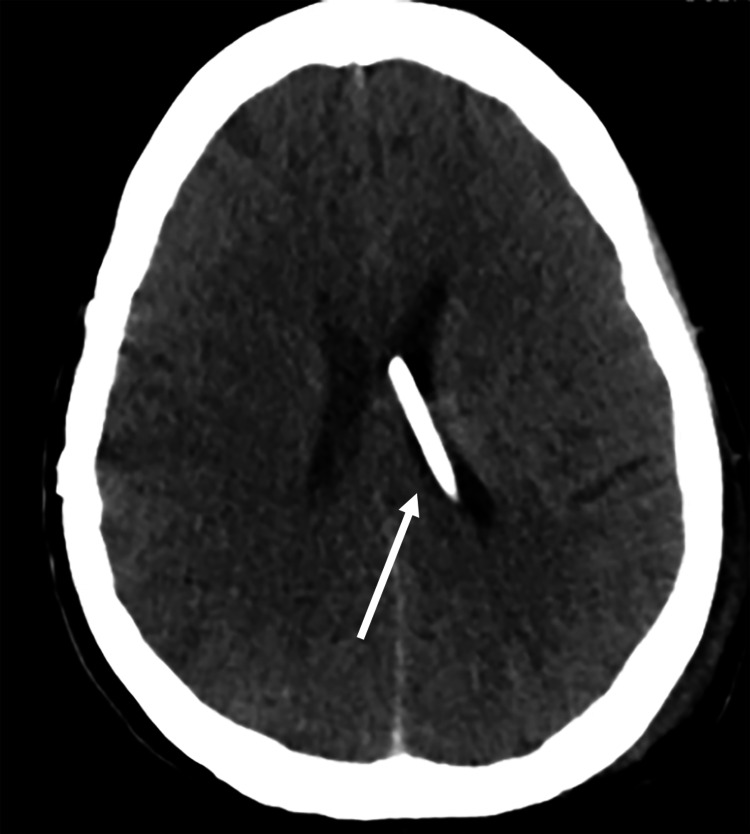
Axial CT without contrast showing good position of the VP shunt catheter with no evidence of acute hemorrhage or ischemia. VP: Ventriculoperitoneal

After an interdisciplinary discussion of the case, it was thought that surgical biopsy would not be necessary to confirm LMD given the positive cerebrospinal fluid analysis, and an urgent ventriculoperitoneal (VP) shunt placement followed by craniospinal irradiation were planned. The VP shunt was placed without complication, and the patient underwent simulation scans for radiation planning on postoperative day one. However, despite being on anti-seizure medication that had been started in the emergency department, the patient had an abrupt neurologic decline later that afternoon with seizure-like activity that required emergent reintubation. A CT scan of the head was unremarkable and showed good position of the VP shunt catheter with no evidence of acute hemorrhage or ischemia (Figure 4). However, an electroencephalogram demonstrated generalized suppression. And on physical examination, the patient met the criteria for brain death. A diagnosis of sudden unexpected death in epilepsy (SUDEP) was concluded, and the patient was transitioned to comfort care. She died shortly thereafter and the family did not wish to pursue an autopsy.

## Discussion

Anaplastic pleomorphic xanthoastrocytoma (APXA) is a rare neoplasm whose characteristics are still being delineated. The APXA was recognized as a unique entity by the WHO in 2016 as a WHO Grade III neoplasm possessing the characteristics of PXA (i.e., cellular pleomorphism, foam cells, and eosinophilic granular bodies with an abundance of reticulin) with a mitotic index of >5/10 per high-powered frame [1,2]. This case demonstrates a remarkably rapid progression from initial diagnosis of APXA to re-presentation with LMD. Prior cases of APXA with extraordinarily early recurrence at one and two months postoperatively have been reported. However, these recurrences were at or near the site of initial resection rather than LMD, and in one patient the APXA likely arose from a lower-grade lesion rather than a primary diagnosis as in our patient [3,4]. The vast majority of reported cases of APXA do not demonstrate such early recurrence. Most experience a recurrence-free period until ~14 months after diagnosis, with some even remaining recurrence-free for two to three years [4].

Although LMD of secondary APXA occurs in up to one-third of adult APXA patients, LMD of primary APXA (as in this case) is extraordinarily rare, although its prevalence is likely underestimated [5]. Of the three adult cases of LMD of primary APXA reported, two showed delayed LMD, and only one presented with LMD at the time of diagnosis [5-7]. The presented case is unique, to the best of our knowledge, for several reasons: 1) the symptoms and findings of hydrocephalus with severe optic disc edema are the first known to be related to APXA with LDM; 2) the three-month interval to death after the initial diagnosis was the shortest known for APXA with LDM; 3) this patient initially presented without LMD, with a subsequent diagnosis just three months after initial diagnosis, serving as evidence for even more rapid LMD in APXA than previously thought; 4) the patient in this case likely died as a result of SUDEP, which has not previously been described as a cause of death in adult APXA with LDM; and 5) after diagnosis of LMD upon re-presentation, the patient experienced multiple strokes secondary to LMD, likely due to the vascular invasion of the tumor, causing narrowing and ischemia of blood vessels. Such findings have not been described in previously reported APXA cases.

Genetically, up to 50 to 78% of PXAs also possess BRAF mutations (most commonly V600E), which correlate with significantly greater overall survival than those that do not [8,9]. However, APXAs possess BRAF mutations less frequently than do nonanaplastic PXAs and have lower five-year survival rates as well (<45% in APXA vs. >80% in PXA) [2,4,9,10]. Although PXAs that occur in childhood have better survival than those that occur in adulthood, a recent study demonstrated that this relationship does not exist in APXAs [10].

The rarity of APXA impedes efforts to generate an ideal treatment regimen. Patients with APXA typically undergo resection followed by adjuvant radiation with or without chemotherapy. However, patients undergoing adjuvant chemotherapy had no better outcomes than those that did not [10]. Although GTR is associated with better outcomes in PXA, patients with APXA who undergo GTR have had similar recurrence rates as those who underwent subtotal resection [10, 11]. Because of the rapid progression seen in our case, we believe earlier craniospinal imaging at the time of APXA diagnosis or shortly thereafter should be pursued to manage disease progression more aggressively with currently recommended adjuvant therapies.

Sudden unexpected death in epilepsy (SUDEP) is sudden, unexpected death in an individual with epilepsy, with or without evidence of a seizure. It excludes documented status epilepticus in which postmortem examination does not reveal a cause of death [12]. With concomitant APXA and LDM with multiple strokes, the described case would be classified as probable SUDEP plus, which indicates that a pre-existing condition could have contributed to the patient’s death; without an autopsy, it was not possible to rule out a cardiac event as a potential contributor [13]. Sudden unexpected death in epilepsy is the leading cause of untimely death in epilepsy, with a 27-fold increase in incidence over sudden death in control populations [14]. For patients with brain tumors, 20 to 45% will present with seizures during the course of the disease, and patients with tumors such as high-grade gliomas experience seizures relatively frequently in the end-of-life phase [15,16]. However, there are very few reports of death in persons with epileptic seizures due to brain tumors, none of which were classified as SUDEP [17]. This adds support to the uniqueness of this case and the aggressive and unpredictable nature of APXA. We believe that the patient’s underlying malignancy and LDM with multiple strokes led to a seizure that likely precipitated her sudden death from either a cardiac insult or a profound hypoxic event.

## Conclusions

This report illustrates the unpredictable biological behavior of APXA and demonstrates the difficulty of adequate disease management. Surgical excision alone appears to be inadequate and should be accompanied by staging scans of the spine followed by radiation. Unfortunately, there was a delay in initiating adjuvant radiation therapy in this patient, which likely contributed to her rapid clinical progression. The short time to onset of LDM in this case demonstrates the need for early and frequent radiological follow-up, which might have improved the clinical outcome in this patient. Additionally, providers should be aware of the rare occurrence of SUDEP in brain tumor patients, as it remains unclear whether this is more common in patients with malignant brain tumors such as our patient, and recognize that it can happen despite patients being on appropriate medications for seizure prophylaxis and in the absence of any clear postoperative complications such as hemorrhage or infection.
